# What adult gastroenterologists should know about young adults with IBD: Navigating transition of care for young adults with IBD

**DOI:** 10.1016/j.hctj.2023.100016

**Published:** 2023-09-01

**Authors:** Sydney Reed, Linda Yoo, Amy Bugwadia, Navin Kariyawasam, Sneha Dave

**Affiliations:** a Generation Patient, United States; b University of Washington, United States; c Stanford University School of Medicine, United States; d University of Toronto, United States

**Keywords:** Health care transition, Emerging adults with IBD, Young adults with IBD, Adult gastroenterologists, Adolescents with IBD

## Abstract

The transition from pediatric to adult care for young adults with inflammatory bowel diseases (IBD) presents unique challenges that can impact the quality of care and patient outcomes. This article summarizes the discussions held during the Roundtable on Young Adults with IBD, focusing on essential information for adult-care providers dealing with patients in transition. Key areas of focus include readiness for transition, effective communication between pediatric and adult providers, transfer of responsibility and care management from caregivers to patients, reforming systems that complicate the transition process, and the importance of support interventions. Addressing these considerations can help healthcare professionals navigate the complexities of transitioning young adults with IBD, ensuring a smoother and more supportive journey into adulthood.

## Introduction

The Roundtable on Young Adults with IBD is a year-long educational community that fosters monthly dialogues between patients and providers, with the aim of improving outcomes for the young adult (YA) IBD patient population. Each month, these discussions revolve around concerns specific to the YA IBD demographic. Our third roundtable session commenced with an introductory presentation led by Linda Yoo, a YA IBD patient, and Dr. Stacy Kahn, Assistant Professor of Pediatrics at Boston Children's Hospital. The presentation addressed the unique challenges in caring for teenagers and YAs with IBD and the complexities of transitioning their care. The presentation was followed by a discussion between a multi-stakeholder group of YA patients with IBD, carepartners, and medical professionals.

Transitioning from pediatric to adult care presents distinct hurdles for YAs diagnosed with IBD. Factors such as timing, coordination among healthcare providers, and gaps in communication can significantly impact the quality of care and patient outcomes during this critical period. This article aims to shed light on essential information that adult-care providers need to be aware of when caring for YA patients navigating this transition process. It is important to distinguish that although the transfer of care is a one-time event, the transition of care is an ongoing process involving the patient, their caregivers, and their pediatric and adult clinical teams. Key areas of focus during the transition process include readiness for transition, emphasizing effective communication between pediatric and adult providers, the transfer of responsibility and care management from caregivers to patients, the challenges posed by systems that complicate the transition process, and the importance of crucial support interventions. By addressing these issues, healthcare professionals can better navigate the complexities of transitioning YAs with IBD and ensure a smoother and more supportive journey into adulthood.

## Readiness for transition

It is important to tailor care to each patient’s context, such as their transitions in education, living with or without parents, or entrance into the workforce. This is crucial for both newly diagnosed patients entering adulthood as well as established pediatric patients transitioning to adult care. One patient shared their experience of being diagnosed with IBD at the age of 20 and feeling uncertain about whether to seek care from a pediatric or adult specialist. The complex and confusing processes of the healthcare system for young adults with IBD can pose a barrier to care. The timing of transitioning to adult care from pediatric care can vary based on healthcare providers, individual circumstances, and insurance coverage. Gray and Maddux (2016) surveyed 141 pediatric IBD care providers and revealed that 21.5 % of patients were transferred to adult care by age 18, 14.5 % by age 21 %, and 11.8 % between ages 22–25, with about one-third having no specific age for transfer^1^. For patients already established with a provider, the age of 18 poses significant challenges for transferring care, as reported by several healthcare providers during our roundtable discussion. Additionally, Philpott and Kurowski (2019) found that 99.3 % of providers faced barriers to transition, including patient reluctance, parental reluctance, provider reluctance, coordination difficulties with adult providers, and a lack of adult providers with pediatric IBD expertise^2^. One provider's insight highlighted the preference to transfer care once patients have achieved a certain level of stability in their adult lives, such as after the patient has completed secondary or post-secondary education. A tailored and patient-centered approach to transitioning ensures a smoother process and provides security and readiness for patients and healthcare providers.

## Communication between pediatric and adult providers

Effective communication between pediatric and adult providers is paramount when transferring the care of YA patients. Many institutions do not have established transition policies, and many adult care offices may not communicate readily or even appreciate the need and importance of such communication when transitioning YA patients with IBD. Communication ensures a smooth transition and continuity of treatment, addressing the specific needs and challenges that arise during this critical period.

A number of healthcare experts have achieved positive outcomes in facilitating patient transitions through the implementation of "joint" medical visits involving the simultaneous presence of both pediatric and adult physicians. Recent research has demonstrated the effectiveness of this approach in transitioning patients with IBD^3^. However, one provider highlighted the challenges of coordinating such visits between two doctors. Alternatively, other healthcare professionals proposed the involvement of nurse practitioners and additional medical transition support staff during these sessions to enhance communication and ensure the seamless transfer of crucial information.

To ensure that patients have landed with new providers and that critical constituent/non-clinical information is carried forward, effective communication channels should be established. Transferring complex information, such as medication regimens, infusion procedures, and other non-clinical aspects, is vital to maintain the patient's well-being and treatment progress. It involves meticulous attention to detail and effective communication channels. This may involve direct communication between pediatric and adult providers through secure electronic platforms, phone calls, or face-to-face meetings. Standardized transfer-of-care protocols and documentation can also aid in conveying critical information accurately and comprehensively. Additionally, involving the patient and their family in the transition process, providing them with resources, and encouraging their active participation can help ensure that important information is conveyed and understood.

## Transfer of responsibility

One YA patient expressed difficulty developing independent communication and self-management skills due to parent involvement during appointments and overall medical care. As IBD is a lifelong condition, pediatric patients eventually need to transition to adult care in order to receive ongoing, age-appropriate medical treatment. However, the challenge lies in the fact that many adolescents nearing the transfer to adult care often lack the necessary self-management skills to effectively navigate their health independently. Not being adequately prepared for the transition to adult care can lead to significant consequences, such as a twofold increase in rates of surgeries and hospitalizations. Thus, it is crucial to address this issue and ensure that YAs with IBD have the skills and support needed to successfully navigate the adult care realm^4^.

It is important to understand that the transfer of responsibility is a sensitive issue that can vary greatly between patient circumstances and family dynamics. Patients whose disease is not well controlled may not be in a position to adequately manage their care. Additionally, parents and/or caregivers of children diagnosed at a very young age might have a more difficult time adjusting to this transition, having played such an active role in the patient’s care for longer periods of time. During the roundtable discussions, healthcare professionals emphasized the significance of customizing the transition process to suit the individual patient needs, but a general consensus emerged urging both providers and caregivers to begin fostering independence and self-management skills in pediatric IBD patients from an early age whenever possible.

As recommended by the American Academy of Pediatrics (AAP), the transition planning process should commence around the age of 12^5^. This early start allows providers sufficient time to involve patients and families in the process, teaching patients the necessary skills to independently and effectively address their healthcare requirements. Offering anticipatory guidance and preparatory information to families regarding the transition planning is likely to alleviate resistance and foreseeable concerns while also promoting increased engagement. Educating families on the reasons for initiating transition planning at an early age on plays a crucial role in fostering a smooth transition process^6^. Understanding this information prepares adult care providers to appreciate the varying levels of preparedness and autonomy that YA patients may have when they enter into adult care and enables them to tailor their approach, providing appropriate guidance to both the patients and their families.

## Reform of current systems that complicate the transition process

The current transition process for patients with IBD is further complicated by the barriers created by the insurance and pharmaceutical industries. These industries often prioritize their profits over the well-being of patients, creating unnecessary obstacles to accessing essential treatments. Pharmacy Benefit Managers (PBMs) add layers of complexity and bureaucratic requirements that providers must navigate in order to ensure patients receive the necessary treatments^7^. Examples specific to IBD patients include delays attributed to prior authorization correlating with delays in biologic initiation and heightened utilization of healthcare resources^8^. These added hurdles not only increase administrative burden but also delay or limit patients' access to critical medications.

Additionally, the pharmaceutical industry's high cost of prescription medicines poses significant financial instability and stress on patients who may face fluctuations in insurance coverage and socioeconomic status as they enter adulthood and the workforce. Current research shows that the USA has a considerably greater number of patents covering biologic drugs compared to the UK and Canada. This higher patent issuance is associated with the delayed entry of biosimilars into the market. A delayed market entry can be attributed to an ineffective patent system, as patent laws aim to provide pharmaceutical companies with a designated period of market exclusivity as a reward for developing therapeutics. However, when pharmaceutical companies exploit the system by relying on numerous low-quality patents that cover minimal contributions, they are taking advantage of a flaw in the system^9^. This manipulation of the patent system restricts the availability of these treatments to patients, posing a hindrance to the transition process and impeding patients' ability to receive appropriate care. Reforming these systems is imperative to prioritize patients' well-being and ensure that unnecessary barriers and limited access to essential treatments do not further burden their transition to adulthood.

## Mechanisms of support as an important intervention

Across multiple roundtable discussions, YA patients have continued to urge providers to incorporate mechanisms of support as critical interventions in treating YAs with IBD rather than additional service options. Peer support, mentorship programs, individual therapy, and other forms of support are essential to address the unique challenges YAs face with IBD. SeveraI IBD patients attested to the vital role that medical professionals play in facilitating one-on-one connections and incorporating support into the overall treatment plan. The experiences shared during this discussion highlight the significant, positive impact these connections can have on disease acceptance and trajectory.

Peer support, in particular, is a valuable yet often underutilized resource. While limited, the literature promotes peer support as a feasible approach to addressing self-management outcomes for AYAs with IBD^10^. This may also help to reach patient populations who may not be well-connected to the healthcare system, such as economically and socially marginalized communities^10^. Currently, only a limited number of endeavors have been undertaken to assess the efficacy of independent peer-support interventions, underscoring the significance of additional research to advance this domain and provide insights for clinical application^11^. Furthermore, it is important to acknowledge that some patients, especially young men with conditions like IBD, may hesitate to join traditional peer-support groups. In such cases, alternative avenues for support should be explored. By recognizing the importance of support mechanisms and adapting them to meet individual patient needs, medical professionals can enhance the care and well-being of YA patients.

## Conclusion

This multi-stakeholder discussion encompassed insight from healthcare professionals, caregivers, and YA patients with IBD. This session shed light on important considerations for adult care providers when dealing with patients transitioning from pediatric to adult care. Key factors include understanding the age of transition, emphasizing effective communication, promoting independence and self-management skills, the need to reform existing systems that complicate the transition process, and incorporating support mechanisms with special emphasis on the role that practitioners can play in facilitating connections between YA patients. By addressing these considerations, healthcare professionals can better navigate the complexities of this transition period, leading to improved outcomes, quality of life, and a more supportive journey into adulthood for YA patients with IBD.

## Funding sources

This work was funded by the 10.13039/100007028Leona M. and Harry B. Helmsley Charitable Trust.

## Declaration of Competing Interest

Sydney Reed, Navin Kariyawasam, Linda Yoo, Amy Bugwadia, and Sneha Dave declare that they have no conflicts of interest.

## Data Availability

No data was used for the research described in the article.
